# Editorial: Real world-evidence in radioligand therapy: above and beyond a tongue twister

**DOI:** 10.3389/fmed.2026.2002519

**Published:** 2026-10-02

**Authors:** Ilaria Grassi, Chiara Maria Grana, Maddalena Sansovini

**Affiliations:** 1 Nuclear Medicine Department, Scientific Institute of Romagna for the Study and Treatment of Tumors (IRCCS), Meldola, Italy; 2 Nuclear Medicine Division, Istituto Europeo di Oncologia, Milan, Italy

**Keywords:** personalized medicine, radioligand, radiopharmaceuticals, real world evidence, theranostics

Radioligand therapy (RLT) has evolved from a highly specialized nuclear medicine approach into an increasingly relevant component of modern oncology. The success of established radionuclide therapies has progressively broadened the theranostic paradigm, while novel targets, radionuclides, treatment settings and strategies for patient selection continue to emerge. In parallel, the growing use of these therapies outside highly selected clinical trial populations has emphasized the importance of evidence capable of reflecting their performance in routine clinical practice.

This Research Topic, *Real World-Evidence in Radioligand Therapy: Above and Beyond a Tongue Twister*, was conceived to explore this evolving landscape. We had the pleasure of bringing together contributions spanning different malignancies, radionuclides and levels of clinical maturity, from the optimization of established radionuclide treatments to emerging therapeutic approaches. Collectively, these articles illustrate both how far the field has progressed and how many questions remain regarding patient selection, treatment optimization and the integration of RLT into increasingly individualized oncologic care.

The original research articles included in this Research Topic provide two different examples of how radionuclide therapy may continue to evolve in clinical practice. Innovation does not necessarily require a new target or radiopharmaceutical; it may also emerge from refining the way established treatments are delivered. Wang et al. investigated the administration of Lugol's solution following radioiodine therapy for remnant ablation in differentiated thyroid cancer. In this preliminary randomized study, structural ablation rates were comparable between groups, while some biochemical outcomes favored Lugol's solution and no clear increase in long-term toxicity was observed. Although the single-center design and limited sample size call for confirmation in larger multicenter studies, these findings illustrate how even long-established radionuclide treatments may still offer opportunities for optimization.

A different therapeutic setting is represented by epidermal therapy with rhenium-188 for non-melanoma skin cancer. Castellucci et al. describe an approach combining high local radiation delivery with limited penetration into deeper tissues. Available clinical experience indicates high response rates, generally favorable cosmetic outcomes and predominantly mild toxicity in appropriately selected patients. At the same time, the current evidence remains largely based on observational and single-center experience. Standardization of dosimetry, optimization of administered dose according to lesion characteristics and prospective comparisons with established treatment options will therefore be important to better define the role of this approach in clinical practice.

Alongside these original contributions, the reviews collected in this Research Topic offer a broader perspective on the evolution of RLT, moving from expanding indications and established radionuclide treatments toward rapidly developing therapeutic strategies and emerging radionuclides.

In our review, Grassi et al. explored RLT for primary brain tumors, including 30 studies on meningiomas and gliomas. Evidence is currently more substantial for meningiomas, whereas RLT in gliomas remains largely experimental. Nevertheless, these experiences are of particular interest because they illustrate the potential of target-based patient selection in diseases for which therapeutic alternatives may be limited. They also expose some of the challenges encountered when RLT moves beyond its best-established indications, including heterogeneous study designs, small patient populations and the need for standardized response criteria and well-designed multicenter trials.

Real-world evidence becomes particularly valuable once radionuclide therapies enter broader clinical use, where patient populations are inevitably more heterogeneous than those enrolled in controlled trials. Song et al. addressed this issue in their meta-analysis of radium-223 in metastatic castration-resistant prostate cancer (mCRPC). Based on 25 observational studies including 8.795 patients, the authors identified several clinical and biochemical variables associated with overall survival and highlighted the relationship between treatment completion and outcome. Their findings emphasize how the effectiveness of radionuclide therapy in everyday practice is closely intertwined with patient selection, disease burden, hematologic reserve, previous treatments and the feasibility of completing the intended therapeutic course.

PSMA-targeted RLT provides perhaps one of the clearest examples of how rapidly a theranostic strategy can evolve. Noamen et al. review the current evidence for PSMA-targeted beta-emitting RLT in prostate cancer and place it within a therapeutic landscape that continues to expand. The field is progressively moving beyond the concept of RLT predominantly as a late-line treatment, with increasing interest in earlier disease settings, therapeutic sequencing and combinations with systemic and local treatments. In this evolving scenario, molecular imaging becomes more than a diagnostic companion: it increasingly contributes to patient selection, assessment of target expression, and characterization of tumor heterogeneity over time. The challenge may therefore be less about identifying a universally superior radiopharmaceutical and increasingly about choosing the most appropriate therapeutic strategy for each biological and clinical context.

At the most innovative end of this continuum, we were particularly pleased to include the contribution by Albano et al. exploring terbium-161-labelled PSMA ligands in mCRPC. Their systematic review and meta-analysis included six studies and 80 patients and reported encouraging disease control and an acceptable safety profile, although the available evidence remains limited and heterogeneous. The additional emission of low-energy electrons by terbium-161 provides an attractive rationale, particularly for microscopic disease. Whether this physical and dosimetric advantage will translate into superior clinically meaningful outcomes remains to be established. Comparative studies, longer follow-up and the incorporation of patient-reported outcomes will be essential before the precise therapeutic positioning of ^161Tb-labelled compounds can be defined.

Taken together, the contributions to this Research Topic reflect a field in transition. From the refinement of established treatments to the exploration of new indications, from real-world experience with consolidated radionuclides to emerging radiopharmaceuticals, they suggest that innovation in RLT should not be understood exclusively as the introduction of a new agent. It may also lie in selecting treatments more intelligently, optimizing their delivery, identifying the patients most likely to benefit and integrating molecular imaging, dosimetry, biological characterization, and clinical information into therapeutic decision-making.

Real-world evidence, systematic reviews, prospective clinical trials and emerging radiopharmaceuticals should therefore not be viewed as competing sources of knowledge, but as complementary components of the same translational pathway. The next phase of development will be defined not simply by the availability of more radiopharmaceuticals, but by our ability to identify the right patient, the right target, the right radionuclide, the right activity and the right time. In our view, this transition from a predominantly radiopharmaceutical-centered perspective toward a genuinely patient-centered model represents one of the most important challenges—and opportunities—for the future of theranostics.

